# Relationship between erectile dysfunction and alexithymia in male patients with implantable cardioverter defibrillators: a cross-sectional study

**DOI:** 10.3389/fpsyt.2024.1327796

**Published:** 2024-10-25

**Authors:** Katharina Ledermann, Claudia Zuccarella-Hackl, Rahel Altwegg, Marc Dörner, Veronica Attanasio, Lisa Guth, Sina Zirngast, Aju P. Pazhenkottil, Anna Menzi, Roland von Känel, Mary Princip

**Affiliations:** ^1^ Department of Consultation-Liaison Psychiatry and Psychosomatic Medicine, University Hospital Zurich, University of Zurich, Zurich, Switzerland; ^2^ Department of Psychology, University of Fribourg, Fribourg, Switzerland; ^3^ Department of Psychology, University of Zurich, Zurich, Switzerland; ^4^ German Center for Neurodegenerative Diseases (DZNE) within the Helmholtz Association, Magdeburg, Germany; ^5^ Cardiac Imaging, Department of Nuclear Medicine, University Hospital Zurich, University of Zurich, Zurich, Switzerland; ^6^ Institute of Molecular Cancer Research, University of Zurich, Zurich, Switzerland

**Keywords:** implantable cardioverter defibrillator, erectile dysfunction, alexithymia, sexuality, adjustment disorder (AjD)

## Abstract

**Background:**

Implantable Cardioverter-Defibrillator (ICD) implantation is a life-saving intervention for individuals at risk of life-threatening arrhythmias. However, the psychosocial impact of ICD implantation extends beyond its cardiovascular benefits, potentially influencing emotional well-being and sexual health. This can lead to erectile dysfunction, which, is often associated with alexithymia. Both erectile dysfunction and alexithymia can significantly affect the psychological well-being of both patients and their partners.

**Aims:**

This study examines the association of erectile dysfunction with alexithymia in patients after ICD implantation. Additionally, we investigate potential moderators of this association.

**Method:**

Patients (N=165) completed self-rating questionnaires: Toronto Alexithymia scale (TAS-20), International Index of Erectile Function (IIEF-5), Adjustment disorder – new module (ADNM-20). Descriptive statistics, correlations, multivariate linear regressions, and moderation analysis were conducted.

**Results:**

The determinants of erectile dysfunction in ICD patients were explored in a regression model explaining 22% of the total variance. The ADNM-20 subscale preoccupation was found to significantly moderate the relationship between the alexithymia subscale externally oriented thinking and erectile dysfunction (R2 = 0.02, p=0.03).

**Conclusion:**

We did not find evidence for a relationship between externally oriented thinking and erectile dysfunction at low to average levels of preoccupation. However, evidence for such a relationship was found at high levels of preoccupation, where more externally oriented thinking was related to more erectile dysfunction. The intersection of alexithymia and erectile dysfunction represents a promising avenue for future research, offering opportunities to unravel the intricate connections between emotional processing and sexual health. Enhancing insights into this relationship could lead to innovative interventions that address the needs of individuals struggling with both conditions, fostering improved emotional expression, intimate relationships, and sexual satisfaction.

## Introduction

The implantable cardioverter-defibrillator (ICD), as a life-saving device, serves as a primary prevention measure for patients at high risk of life-threatening ventricular arrhythmias or sudden cardiac death and as a secondary prevention option for those who have survived such critical cardiac conditions (1). As a result, ICD implantation has become a well-established treatment for various cardiac conditions, including coronary heart disease (e.g. prevention of sudden cardiac death following myocardial infarction), advanced heart failure, and inherited heart diseases, such as hypertrophic cardiomyopathy or arrhythmogenic right ventricular cardiomyopathy (2). While ICDs provide essential protection against life-threatening cardiac arrhythmia, they can impact various aspects of patients’ lives, including their sexual functioning (3).

Sexual dysfunction, which includes difficulties or disturbances in sexual desire, arousal, or satisfaction that interfere with a person’s ability to engage in satisfying sexual activities, is common among patients with an ICD (3–5). A study involving 443 male and female ICD patients found that 35.4% of patients were unable to engage in sexual activities after ICD implantation. Similarly, while the majority of patients reported having the ability to engage in sexual activity (64.6%), approximately half of them chose to avoid it (4). Another study among 415 male ICD patients revealed that 70% experienced erectile dysfunction (ED), 57.9% had orgasmic dysfunction, 82.8% experienced reduced sexual desire, 85.8% had problems with intercourse satisfaction, and 76.9% reported overall satisfaction problems. Patients with an ICD implanted for primary prophylactic indication showed higher rates of ED compared to those with an ICD implanted for secondary prophylactic indication. Moreover, the study found that anti-tachycardia pacing (ATP) therapy, but not shocks, was associated with an increased risk of ED (5). Patients attribute this to apprehension, fear, and concerns that sexual activity might trigger a cardiac event or ICD shock (3–6). Research suggests that a substantial proportion of patients hold misconceptions regarding the safety and effectiveness of their ICD during sexual activity (3). According to the guidelines issued by the American Heart Association (AHA), the majority of patients can safely resume sexual activity. However, caution is advised for individuals with sub-optimally controlled arrhythmias or those who experience ventricular arrhythmias triggered by moderate physical activity (7, 8). Nevertheless, the specific factors associated with ED remain unclear.

Alexithymia, a psychological trait characterized by difficulties in recognizing and expressing emotions, has been a topic of growing interest in the realm of sexual health research (9). Introduced by Nemiah and Sifneos in the early 1970s, alexithymia is a multidimensional construct that encompasses a set of affective and cognitive characteristics (10, 11). It describes deficits in cognitive processing of emotions and challenges in emotion regulation. Individuals with alexithymia struggle to a) identify and differentiate emotions from bodily sensations, b) communicate emotions to others, and c) engage in imaginal and fantasy activities (12). Several studies suggest that alexithymia is relatively stable over time, reinforcing the notion that it is a stable personality trait. For instance, Bagby, Parker and Taylor (13) argued that alexithymia reflects a long-term personality characteristic rather than a transient state. Contrary to the perspective of stability, other studies indicate that alexithymia can change over time, particularly in response to therapeutic interventions and significant life events. For instance, De Gucht et al. (14) found that alexithymia scores decreased following psychotherapy in patients with functional somatic syndromes, suggesting that targeted therapeutic approaches can lead to significant changes in alexithymic traits. Moreover, Saarijärvi et al. (15) reported that alexithymia levels can fluctuate in response to changes in psychological well-being. Their study showed that improvements in depression and anxiety were associated with reductions in alexithymia, indicating that emotional and psychological states can influence the expression of alexithymic traits. In conclusion, alexithymia may be best understood as a relatively stable trait with the potential for modification under certain conditions. Further research is needed to delineate the specific factors that contribute to its stability and malleability, and to develop effective interventions for those affected by high levels of alexithymia.

The relationship between alexithymia and ICD patients can be attributed to several factors. Firstly, the emotional regulation difficulties inherent in alexithymia can lead to increased psychological distress (16), which in turn exacerbates the perception of pain and discomfort associated with ICD. This heightened pain perception can negatively impact sexual desire, arousal, and overall sexual satisfaction. Additionally, the perception of pain and discomfort experienced by ICD patients can create a negative feedback loop, where the physical symptoms of the condition are amplified by the psychological burden of alexithymia. This interplay between psychological and physical factors further complicates the sexual health of ICD patients. Greater manifestation of alexithymia has been found to be linked to various sexual health issues (17, 18). On the one hand, studies have demonstrated associations between alexithymia and conditions such as ED (17–19), hypoactive sexual desire (17), and premature ejaculation (19). Additionally, research involving nonclinical female samples has revealed associations between alexithymia and lower sexual desire (19) and increased sexual dissatisfaction (9, 20), and reduced frequency of vaginal intercourse (21). On the other hand, associations between alexithymia and engaging in risky sexual behaviors have been found (22). The Swiss report authored by Madioni and Mammana revealed notable levels of alexithymia in patients diagnosed with both ED and hypoactive desire disorder (19). In contrast, a clinical group comprising individuals with orgasm disorders, including premature ejaculation and inhibited male orgasm, did not exhibit similarly elevated alexithymia scores (19). As patients with ICDs are more likely to face heightened psychological distress due to their cardiac condition and device implantation (1), it is crucial to investigate the potential relationship between ED and alexithymia in this population. To the best of our knowledge, there is no study that explored the interplay between ED and alexithymia in ICD patients. Previous studies have largely focused on the psychological impact of living with an ICD, including anxiety, depression, and quality of life while neglecting the potential influence of alexithymia on sexual function (1).

This study aims to bridge this critical gap in the literature by comprehensively examining the relationship between ED and alexithymia in ICD patients. The study will explore the prevalence of ED and alexithymia in a male population living with an ICD and investigate whether there is a significant association between these two constructs. Additionally, we will explore potential underlying factors that may moderate this relationship, such as ICD concerns, adjustment disorder, depressive and anxiety symptoms. The findings from this study hold the potential to inform healthcare providers, psychologists, and cardiologists about the psychosocial aspects of living with an ICD. Such insights can guide the development of tailored interventions to address sexual health concerns and alexithymia in these patients, ultimately contributing to a more comprehensive approach to their overall well-being and enhancing their quality of life.

## Methods

### Study procedure and participants

This was a secondary analysis of the research project “Implantable cardioverter-defibrillator and development of an adjustment disorder”, a national, single center, exploratory cross-sectional study with a follow-up at 6 months. The objective of the study was to investigate the difference in adjustment disorder in ICD patients with versus without one or multiple shocks and electrical storms at admission. The project was carried out in accordance with the Declaration of Helsinki and approved by the Ethics Committee of the Canton of Zurich (BASEC 2019- 01948). All participants enrolled provided oral informed consent prior to the start of the study. Data collection took place from January 2020 to September 2023. Participants were recruited at the University Hospital Zurich (USZ) in the Department of Cardiology at the outpatient tertiary Cardiac Arrhythmia Division. Participants were aged 18 years or older with an implanted ICD or any other implantable device with an ICD-function (e.g. Cardiac Resynchronization Therapy). Exclusion criteria were insufficient knowledge of German language in reading and understanding, and decision to exercise the right to forgo information on clinically relevant findings. Patients attended the USZ for their regular half-yearly ICD check-up appointment. All participants received written and oral information on the study. After agreement for study participation, participants completed questionnaires at home and resent them to the study team. For the participation in the study, participants did not receive any reimbursement.

### Psychometric assessment

#### Adjustment disorder – New module 20

Adjustment disorder symptoms (AjD) were measured with the validated German version of the Adjustment Disorder – New Module 20 (ADNM-20) (23). The ADNM-20 is a self-reported questionnaire to measure AjD with the new ICD-11 criteria. It consists of two parts: a stressor list and an item list. The stressor in this study was the burden in relation to the ICD. The 20 items are rated on a 4-point Likert scale (1 = “never”, 4 = “often”) indicating how often a person has experienced different symptoms of an AjD. The ADNM-20 questionnaire comprises six subscales: preoccupation (4 items), failure to adapt (4 items), avoidance (4 items), depressive mood (3 items), anxiety (2 items), and impulse disturbance (3 items). Since preoccupation and failure to adapt represent the central symptoms of the new AjD diagnosis, these two subscales can be combined into a single scale referred to as core symptoms (AjD-C). The subscales that detail additional symptoms can also be consolidated into one scale (AjD-AS). The severity of symptoms can be assessed using either the total score of all items in the ADNM-20 (ADNM-20 sum score). The internal consistency was α = .95, the estimated reliability for the two ADNM subscales was good (preoccupation with stressor α = 0.88, failure to adapt α = 0.84).

### International index of erectile dysfunction

ED was measured with the German version of the shortened 5-item version of the International Index of Erectile Function (IIEF-5) (24). Items are rated on a 6-point Likert scale (0 = “not at all”, 5 “quite always or always”), with higher scores indicating better erectile function. The total score ranges from 0 to 30. Scores between 0 to 10 indicate severe ED, scores between 11 to 16 indicate moderate ED, scores between 17 to 25 indicate mild ED, and scores between 26 to 30 indicate no ED. The internal consistency of the IIEF-5 sum score was α = .95 for ED in our study.

### Toronto alexithymia scale

The German adaptation of the 20-item Toronto Alexithymia Scale (TAS-20) was used to assess deficiencies in comprehending, processing, and articulating emotions (25). The TAS-20, a self-report questionnaire, evaluates three facets of alexithymia: (1) Difficulty Identifying Feelings (DIF) - indicating uncertainty about one’s emotion’s origins; (2) Difficulty Describing Feelings (DDF) - expressing struggles with verbalizing emotions, as people suggest; and (3) Externally Oriented Thinking (EOT) - revealing difficulties in disclosing inner emotions, even to close friends. Items were rated on a 5-point Likert scale, ranging from 1 (strongly disagree) to 5 (strongly agree). Negative items (4, 5, 10, 18, and 19) are reverse-scored. The composite alexithymia score combines responses from the three subscales. Categorization is based on cut-off scores: 51 or less for ‘non-alexithymia,’ 61 or more for ‘alexithymia,’ and scores between 52 and 60 indicate ‘possible alexithymia’ (13). The German version’s validity was demonstrated through convergent and clinical assessments (26). The internal consistency for the TAS-20 sum score was α = .81 in our study (DIF: α = 0.84, DDF α = 0.63, EOR = 0.46).

*ICD Patient Concerns Questionnaire*: ICD-related concerns were assessed using the 8-item Implantable Cardioverter Defibrillator Concerns Questionnaire (ICDC) (27). The ICDC measures the number and severity of patient concerns related to the ICD on a five-point Likert scale. The ICDC has been found to be valid and reliable in multiple populations.

*Depressive Symptoms:* The German version of the Patient Health Questionnaire (PHQ-8) was applied (28, 29). The PHQ-8 is a self-administered questionnaire to measure depressive symptoms. Cronbach Alpha in our sample was α = 0.82.

*Anxiety Symptoms*: GAD-7: Generalized Anxiety Disorder 7 (GAD-7) assesses all levels of anxiety symptoms (30). The GAD-7 is a self-report questionnaire for screening and severity measuring of generalized anxiety disorder (GAD). Cronbach Alpha in our sample was α = 0.9.

### Statistical analysis

Descriptive statistics for patient characteristics and further inferential statistical analysis were conducted using SPSS 29.0 for Windows (SPSS Inc., Chicago, IL, USA). All statistical tests were two-tailed and the significance value was set to an α = 0.05. Kolmogorov-Smirnov tests indicated that all TAS-20 scores, IIEF-5, ADNM-20, ICDC, GAD-7and PHQ-8 scores were not normally distributed. Therefore, Spearman correlations were calculated to assess bivariate associations between variables (TAS-20 sum and subscales, IIEF-5, ADNM-20, ICDC, GAD-7, PHQ-8). Potential moderating effects of ADNM-20 scale, ICDC, GAD-7 and PHQ-8 on the association between the total and subscales of the TAS-20 and sexual dysfunction were calculated using the PROCESS macro for SPSS (version 4.2) (31). PROCESS is used to examine moderation and mediation, with the benefit of saving time by automating some steps (31). In the moderation analysis using PROCESS (model 1), TAS-20 sum score, TAS-20 subscales DIF, DDF, EOT were entered as the predictor variable (X), the dimensional IIEF-5 score was the outcome variable (Y), and ADNM-20, ICDC, GAD-7 and PHQ-8 scores added as the moderator variable (M) in separate models. The assumption of normally distributed residuals is met for all variables. Given the association with the outcome variable, age was included as a covariate. *Post-hoc* analyses using simple slope analyses and the Johnson-Neyman method were used to further investigate any significant moderation effects observed. Further, regression centering was performed. Regression output showed no concern for multivariate outliers using Cook’s distance, multicollinearity or violation of homoscedasticity assumption constructing a residuals plot.

## Results

### Patient’s characteristics

**Table 1** depicts the characteristics of the participants included and the missing data. The participants had a mean age of 60 years, were mostly married, retired, and lived with someone. The mean age at ICD implantation was 55 years, and most of the participants had experienced a shock delivery after more than one year of ICD implantation.

**Table 1:** Sociodemographic and clinical Characteristics of study 320 male participants.

Variables	Overall (n=320)
Age, years, M (SD)	n=165 58.9 (12.97)
Marital status, n (%)	n=165
married	116
divorced	23
widowed	5
single	21
Living situation, n (%)	n=310
alone	65 (20.3)
living with someone	245 (76.6)
Work status, n (%)	n=165
full-time	59
part-time	20
unemployed	13
retired	73
Age at the ICD implantation, M (SD)	n=165 56.24 (13.82)
Experience of shock delivery, n (%)	n=165
yes	70
no	95
Occurrence of the first shock delivery, n (%)	n=70
in the first month	3
within the first 1 to 6 months	13
within the first 6 to 12 months	14
> 12 month	35
never	5
Clinical characteristics:	
ICDC total score, M (SD)	n=165 6.69 (7.21)
PHQ-8 total score, M (SD)	n=165 4.29 (4.37)
GAD-7 total score, M (SD)	n=165 3.37 (4.44)
IIEF-5 total score, M (SD)	n=288 14.17 (11.03)
TAS-20 sum, M (SD)	n=165 43.30 (11.25)
TAS-DIF subscale, M (SD)	n=165 12.18 (5.33)
TAS-DDF subscale, M (SD)	n=165 10.90 (3.54)
TAS-EOT subscale, M (SD)	n=165 20.36 (5.05)
ADNM-20 p subscale, M (SD)	n=165 8.24 (3.11)
ADNM-20 f subscale, M (SD)	n=165 7.18 (3.13)
ADNM-20 total score, M (SD)	n=151 38.01 (14.45)

ICDC, ICD concerns questionnaire; PHQ-8, Patient Health Questionnaire; GAD-7, General anxiety questionnaire; IIEF-5, International Index for erectile dysfunction. TAS-20 sum, Toronto alexithymia scale total score; TAS DIF, Toronto alexithymia scale- difficulties identifying feelings subscale; TAS-DDF, Toronto alexithymia scale – difficulties describing feelings subscale; TAS EOT, Toronto alexithymia scale – externally oriented thinking subscale; ADNM-20 p, Adjustment Disorder – New Module 20 (ADNM-20) preoccupation subscale; ADNM-20 f, Adjustment Disorder – New Module 20 (ADNM-20) failure to adapt subscale; ADNM-20 sum, Adjustment Disorder – New Module 20 (ADNM-20) sum of preoccupation and failure to adapt subscale.

### Correlations between erectile dysfunction and alexithymia

IIEF-5 total score correlated negatively with the TAS-20 subscale EOT (r= -0.14; p = 0.017). No correlation was found for the subscales DIF and DDF. The negative association between IIEF total score and the TAS-20 total score was not significant (r = -0.11, p = 0.055). Additionally, the TAS-20 subscales DIF, DDF, and EOT and the TAS-20 total scores were positively associated with the ADNM-20 subscales p and f, and with the ADMN-20 sum score (see **Table 2**). Furthermore, TAS-sum and TAS-20 subscales DIF and DDF were positively associated with ICDC. PHQ-8 and GAD-7 correlated positively with TAS-20 sum score and all three subscales (p<0.001).

**Table 2:** Descriptive statistics and intercorrelations.

Variable	IIEF-5	TAS-20 sum	TAS DIF	TAS DDF	TAS EOT	ADNM-20 p	ADNM-20 f	ADNM-20 sum
IIEF-5	(-)	-.10	-.02	-.08	-.13**	.09	.10	.06
TAS sum	-.10	(-)	.75**	.85**	.73**	.37**	.36**	.39**
TAS DIF	-.02	.75**	(-)	.61**	.23**	.52**	.51**	.56**
TAS DDF	-.08	.85**	.61**	(-)	.49**	.27**	.25**	.26**
TAS-EOT	-.13**	.73**	.23**	.49**	(-)	.70	.35	.06
ADNM-20 p	.10	.37**	.52**	.27**	.41	(-)	.73**	.91**
ADNM-20 f	.10	.36**	.52**	.25**	.23	.73**	(-)	.87**
ADNM-20 sum	.06	.39**	.56**	.26**	.35	.91**	.87**	(-)

N= 165 IIEF-5, International Index for erectile dysfunction. TAS-20 sum, Toronto alexithymia scale total score, TAS DIF, Toronto alexithymia scale- difficulties identifying feelings subscale; TAS-DDF, Toronto alexithymia scale – difficulties describing feelings subscale; TAS EOT, Toronto alexithymia scale – externally oriented thinking subscale; ADNM-20 p, Adjustment Disorder – New Module 20 (ADNM-20) preoccupation subscale; ADNM-20 f, Adjustment Disorder – New Module 20 (ADNM-20) failure to adapt subscale; ADNM-20 sum, Adjustment Disorder – New Module 20 (ADNM-20) sum of preoccupation and failure to adapt subscale. **significance level p<0.01.

*significance level p<0.05, **significance level p<0.01.

### Determinants of erectile dysfunction

**Figure 1:**
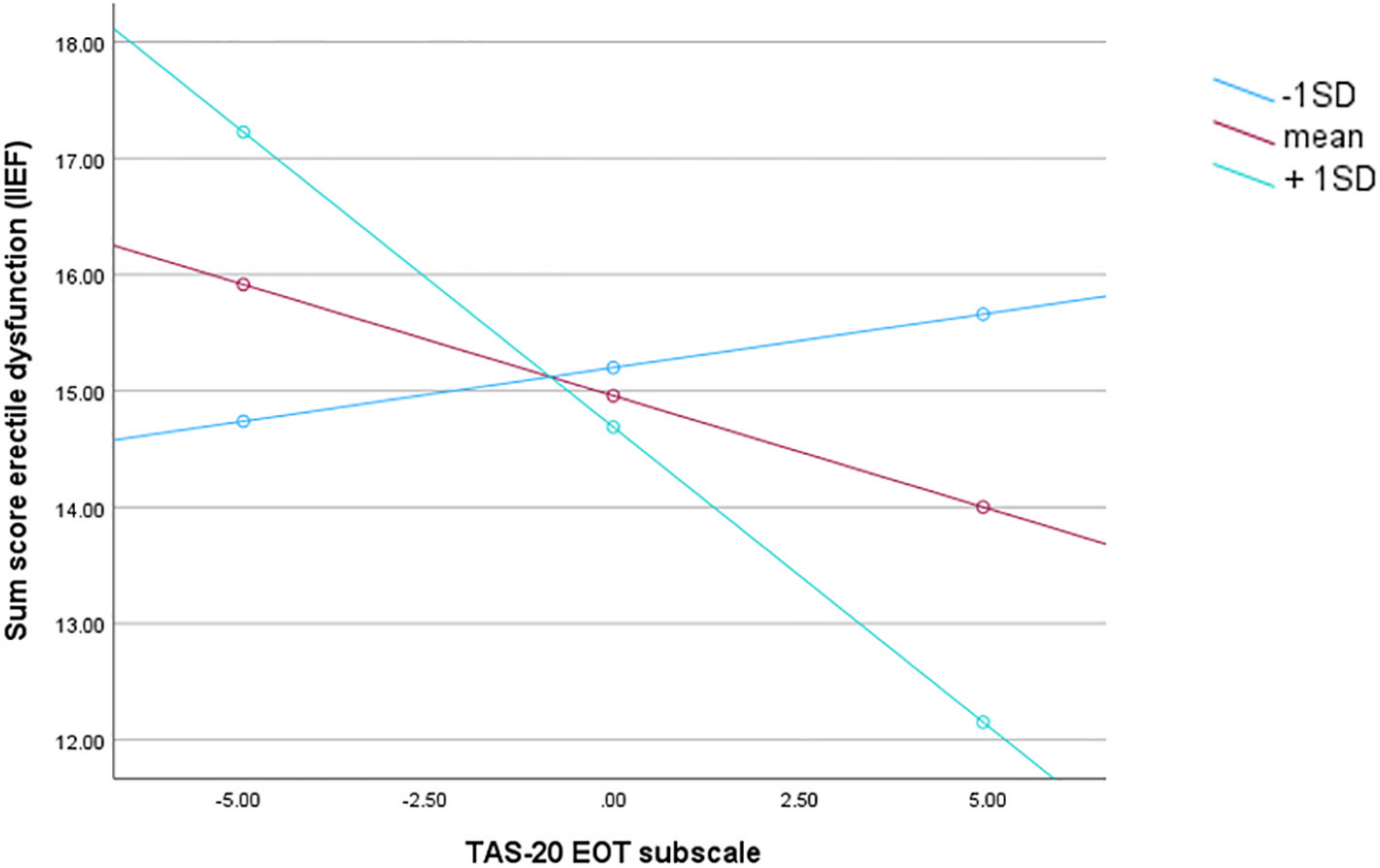
Illustration for the simple slopes of alexithymia (EOT) predicting erectile dysfunction for 1 SD below the mean adjustment disorder symptoms preoccupation (ADNM-20 preoccupation), the mean adjustment disorder symptoms preoccupation (ADNM-20 preoccupation), 1 SD above the mean adjustment disorder symptoms preoccupation (ADNM-20 preoccupation). Note: lower score on the y-axis means more erectile dysfunction.

The determinants of ED in ICD patients were explored in a regression model explaining 21% of the total variance (*F*4.161)=10.79, p < 0.001, *R^2^* = 0.21. The TAS-20 subscale EOT was significantly associated with IIEF-5 (rs(165) =(-0.13), p = 0.02). The total TAS-20 and the other two subscales were not associated with IIEF-5 (*p* > 0.5). The simultaneously entered ADNM-20 subscale preoccupation was found to significantly moderate the relationship between TAS-20 EOT and IEFF-5 (*R^2^* = 0.02, *p* = 0.03) (**Table 3**). The ADNM-20 subscales failure to adapt and the sum of preoccupation and failure to adapt had no influence on the relationship between IIEF-5 and EOT. We probed the interaction in two ways using simple slopes and the Johnson-Neyman method high (+1 SD above the mean of the ADNM-20 subscale preoccupation), medium (mean of the ADNM-20 subscale preoccupation) and low (-1 SD below the mean of the ADNM-20 subscale preoccupation) (**Table 4**). The Johnson-Neyman method was applied to identify the threshold of the moderator (M) preoccupation where the association between the predictor X (EOT) and the outcome Y(IIEF-5) transitions between statistical significance and non-significance (32). Simple slope analysis revealed that when the ADNM-20 subscale preoccupation was higher, the association between IIEF-5 and EOT became increasingly negative. In other words, the impact of EOT on IIEF-5 differed in patients with high (1SD above the mean, *β* = -5.1, p < 0.001) levels of ADNM-20 subscale preoccupation to those with low and medium levels of ADNM-20 preoccupation. Using the simple slope method, at low and medium levels preoccupation the relation between EOT and IIEF-5 was not statistically significant (p > 0.6); however at high levels (*t*(166) = -2.30, *p* = 0.02) ADNM-20 preoccupation was a significant moderator of the association between IIEF-5 and EOT (see **Figure 1**). The moderator value defining the Johnson-Neyman significance region was an ADNM preoccupation value of (70.48% below; 29.51% above), confirming the results of the first method used.

**Table 3:** Parameter estimates and model prediction for determinants of sexual dysfunction of male patients after ICD implantation.

R	R2	MSE	F	df1	df	p
0.45	0.21	96.82	10.79	4.0	161	0.01
Model						
	β	SE	t	p-value	LLCI	ULCI
Constant	25.60	8.26	3.09	0.01	9.28	41.92
TAS-EOT	.46	.36	1.28	.19	-.24	1.17
ADNM-20p	1.79	.97	1.84	.06	-.12	3.71
Interaction	-.09	.04	-2.04	.04**	-.18	-.21
Age	-.33	.05	-5.75	.01**	-.44	-.21

The table presents the significant interaction term of TAS-20 EOT and ADNM-20 preoccupation subscale on determinants of sexual dysfunction measured with IIEF-5. TAS-20 EOT; Toronto alexithymia scale – externally oriented thinking subscale ADNM-20 p: Adjustment Disorder – New Module 20 (ADNM-20) preoccupation subscale; *significance level at p<0.05, **significance level at p<0.01.

**Table 4:** ADNM-20 preoccupation (at levels of low/medium/high) as a moderator between TAS-20 EOT and IIEF.

	β	SE	t	p-value	LLCI	ULCI
-1 SD=4.0	.09	.21	.43	.66	-.32	.51
M=7.1	-.19	.15	-1.21	.22	-.51	.12
+1 SD=10.5	-.51	.22	-2.3	.02**	-.95	-.07

The table presents the results of the simple slopes analysis. ADNM-20 preoccupation was a significant moderator of the relationship between TAS-20 EOT at high levels of ADNM-20 preoccupation and IIEF. *significance level at p<0.05, **significance level at p<0.01.

## Discussion

This study investigated the association of alexithymia and ED among men after ICD implantation. 61.73% of men in our sample reported some form of ED after ICD implantation. In total, 40.81% of the participating men reported moderate to severe ED following ICD implantation whereas only 38.27% did not report any ED. This aligns with the findings of a recent study from Denmark, which utilized the same ED instrument as ours and reported over 70% ED in a sample of ICD patients (5). Older studies utilizing different instruments to assess ED reported prevalence rates of 56% (6, 31) and 57% (6). Additionally, the purpose of ICD placement whether prophylactic or not, appeared to have an impact on the development of ED (5). Regarding the relationship between alexithymia and ED in ICD patients, we found a negative association between the alexithymia subscale externally oriented thinking and severity of ED. Furthermore, we found that this relationship was moderated by the factor “preoccupation” of the adjustment disorder scale. This means that we did not find evidence for a relation between externally oriented thinking and erectile dysfunction at low to average levels of preoccupation, while we found evidence for such a relation at high levels of preoccupation, where more EOT was related to more ED. The association between the total alexithymia score and severity of ED did just not reach the significance level, but also showed a negative tendency in the sense that the more alexithymic someone was, the more severe ED was. Additionally, alexithymia correlated with all three sub-scales, as well as with the severity of the adjustment disorder and its factors, preoccupation and failure to adapt. When ED is divided into severity levels, the following results were observed: Severe ED correlated with alexithymia and all its sub-scales. Moderate ED only correlated with the difficulty describing feelings. In patients with mild ED or no erectile difficulties at all, no association with alexithymia could be established. This suggests that the more alexithymic an individual is, the more they also suffer from sexual dysfunction. This is in line with previous studies that have demonstrated a connection between alexithymia and ED in otherwise healthy men, as well as the potential impact of ICD implantation on sexual function (18). However, this is to our knowledge the first study to integrate these constructs and show that alexithymia may be associated with sexual dysfunction in ICD patients. Furthermore, the novel and unexpected finding is that externally oriented thinking, in particular, is related to the severity of ED. Additionally the moderation of the relationship between externally oriented thinking and the severity of ED by preoccupation is a new and unique aspect. There is limited literature on this topic, as we employed a novel adjustment disorder instrument in our study. One possible explanatory factor could also arise from the notion that individuals with higher levels of alexithymia may possess fewer interoceptive abilities. Previous studies have identified a relationship between greater alexithymia and diminished interoceptive awareness in men with ED. Furthermore, a connection between interoceptive awareness and delayed ejaculation has been reported (18). Given the close connection between emotional well-being and sexual satisfaction, a lack of emotional awareness could lead to challenges in comprehending and managing emotions during intimate moments, thus affecting an individual’s capacity to fully engage in sexual experiences. Furthermore, it is worth noting that alexithymia imposes limitations on interpersonal relationships. Consequently, it could lead to decreased intimacy, and this reduced intimacy might serve as a mechanism through which alexithymia influences poorer sexual functioning. The limited imaginative involvement observed in individuals with alexithymia could potentially lead to a dearth of sexual fantasies, a component widely acknowledged as fundamental in human sexuality. Consequently, the findings may suggest that among ICD patients experiencing sexual dysfunction, disturbances in emotional regulation are particularly intertwined with a cognitive orientation towards external stimuli. This specific facet of alexithymia, coupled with difficulties in describing feelings (DDF) to others, aligns with the French psychosomatic concept of «pensée opératoire» (operational thinking) – a cognitive style characterized by a preference for external details over emotions, fantasies, and other aspects of inner experience (33). This tendency could impose constraints on interpersonal relationships, potentially contributing to reduced intimacy. This diminished intimacy might represent a plausible pathway through which alexithymia becomes connected to poorer sexual functioning. Further investigation is essential to attain a clearer understanding of this issue.

Our findings additionally demonstrated that the relationship between ED and the alexithymia subscale externally oriented thinking is moderated by the variable «preoccupation» from the new adjustment disorder symptom scale. This shows specifically that the stronger the preoccupation of an individual, the more pronounced the relationship between ED and externally oriented thinking becomes. Externally oriented thinking is related to a cognitive style in which an individual tends to focus their attention and thoughts predominantly on external stimuli, events or circumstances in the outside world, rather than on their internal thoughts, emotions or bodily sensations. It often involves a decreased awareness or preoccupation with one’s own inner experiences and a heightened emphasis on the external environment. This style of thinking is often associated with reduced introspection and self-awareness (34).

In light of these results, it is pertinent to consider earlier studies that have suggested a connection between patients following ICD implantation and adjustment issues, often leading to the manifestation of anxiety and a heightened internal focus (35, 36). This heightened focus on internal sensations can be attributed to the anxiety, where the heart region is monitored more closely, and activities that induce an increase in heart rate may lead to anxiety or avoidance. Within this context, it is plausible that the aspect of externally oriented thinking might operate as a constructive coping mechanism, potentially counteracting the onset of ED. This is also evident in reports of patients frequently reducing or ceasing sexual activity, despite reassurance that sexual activity is not harmful (18). Both patients and their spouses often have concerns that engaging in sexual activity might trigger the ICD device (6). Previous studies have reported instances of activity avoidance and shock anxiety stemming from the planted device, with 35.4% of patients being unable to engage in sexual activity. Additionally, 48.5% of patients reported avoiding sexual activity regardless of their ability to perform it (4). While the fear of receiving a shock was a significant reason for avoidance, the apprehension over raising heart rate often took precedence. For instance, 3.4% of patients mentioned avoiding sex because due to fear of receiving a shock, whereas 6.3% refrained due to concerns about an elevated heart rate (4). The relationship between ED and ICDs is an intriguing and relatively unexplored area within the realm of cardiovascular medicine and sexual health. While limited research has been conducted on this specific interaction, the interplay between these two domains is influenced by physiological, psychological, and hormonal factors that underlie both conditions (34). On a physiological level, ED is often a consequence of impaired blood flow to the penile tissue, resulting in an inability to achieve or sustain an erection suitable for sexual activity (37). Cardiovascular health plays a pivotal role in this process, as conditions such as hypertension, atherosclerosis, and diabetes – commonly associated with the need for ICD therapy – can contribute to vascular dysfunction (37). ICDs are implanted to manage arrhythmias and sudden cardiac death, thus targeting the same cardiovascular risk factors that can influence ED. Consequently, ED and CVD share identical principal cardiovascular risk factors and pathophysiological pathways (38). ED also seems to be a major contributing factor to the discontinuation of, and poor adherence to cardiovascular therapy (39). Finally, the pharmacological management of ED, in addition to the well-characterized localized beneficial effects on ED, also seems to have favorable systemic effects on the cardiovascular system (40). Psychological factors also play a substantial role in the development and progression of ED. Living with an ICD can introduce various psychological stressors, such as anxiety about device shocks, alterations in body image, and the constant awareness of one’s cardiac condition (6). These stressors can impact sexual self-esteem and body confidence, potentially contributing to ED. Moreover, the fear of physical exertion triggering arrhythmias might lead to avoidance of sexual activity, further exacerbating sexual dysfunction (5). Furthermore, the physiological effects of ICDs on neural pathways and hormonal regulation of sexual function remain an intriguing avenue for investigation. ICDs generate electrical impulses to monitor and regulate cardiac rhythms. The potential interaction between these electrical signals and the neural pathways that govern erectile responses is a topic that warrants further exploration. In summary, our initial exploration into the association between alexithymia and ED in patients with ICD implants highlights a notable connection between alexithymia and specific sexual issues. Viewed in this light, certain sexual symptoms could be construed as manifestations of broader somatization disorders. Challenges related to emotions and affect may hinder the formation of intimate connections. However, it is crucial to acknowledge that the relationship between ED and alexithymia is intricate and can be influenced by various factors, including cultural and individual differences.

### Clinical implications

Recognizing the profound psychological impact of ICD implantation on sexual health is crucial for healthcare providers. Addressing sexual issues in cardiac patients requires not only an interdisciplinary approach but also a high degree of sensitivity. Discussing sexual problems that consider not only the cardiac aspect but also the emotional and psychological well-being of patients after an ICD implantation should be integrated into routine visits. Open and empathetic communication between healthcare providers, patients, and their partners can facilitate a better understanding of the potential challenges and promote strategies for coping and maintaining healthy intimate relationships. Therapies that focus on improving emotional awareness and communication skills, such as psychotherapy and cognitive behavioral therapy can be beneficial in helping individuals with alexithymia develop healthier emotional connections and improve their sexual experiences (18). Mindfulness training has been shown to have a positive effect on increasing interoceptive awareness and alexithymia as it encourages individuals to observe bodily sensations, thoughts and emotions without getting entangled in them (18). Additionally, addressing any underlying psychological or medical factors contributing to sexual dysfunction is also crucial for effective treatment. The complex interrelation between cardiovascular health and sexual function is underscored by the intricate vascular and neural networks that govern both systems (37). Shared risk factors, such as diabetes, hypertension, and atherosclerosis, contribute to the development of both cardiovascular disease and ED (38). Considering the intimate connection between these two domains, it is plausible that interventions aimed at improving cardiac health may inadvertently influence sexual function.

### Limitations

The limitations for this study include the use of cross-sectional data where changes in measures of sexual activity and sexual concerns over time were not collected and causal relationships between variables cannot be made. We therefore cannot rule out preexisting sexual dysfunction. Additionally, the data for this subsample analysis were collected using self-report measures with the potential of reporting bias such as social desirability. The homogeneity of the final sample (primarily Caucasian) may limit the generalizability of our findings. Since we focused on the comparison between a clinical group and on the correlation between alexithymia and ED severity, a further investigation with a normal control group could be interesting to increase the comprehension of this phenomenon, although previous reports suggested that alexithymia level is significantly higher in patients with ED than in the normal sample (39). A limitation of our study is the estimation of 24 moderation effects, which introduces the possibility of a multiple testing problem. This increases the risk that some of the statistically significant moderation effects identified may be false positives. While we have taken care to ensure the robustness of our analyses, the multiple comparisons made in this study could inflate the likelihood of Type I errors. Future research should consider replication of these findings with a focus on correcting for multiple testing to confirm the validity of the moderation effects observed. Furthermore, we cannot rule out the influence of cardiovascular drugs on our results. The best-studied cardiovascular drugs in terms of their effects on ED are the antihypertensive drugs because of the strong interaction between hemodynamic parameters and ED (40). Some classes of antihypertensive drugs have neutral or even beneficial effects on sexual dysfunction, whereas others have been shown to have detrimental effects on sexual health (40). In general, older antihypertensive drugs (first and second generation Beta Blockers, diuretics and centrally-acting agents such as methyldopa) tend to have a negative effect on erectile function, whereas newer medications have either a neutral effect or beneficial effects (nebivolol and Angiotensin receptor blockers) (40). Another limitation is that certain patients received antidepressant agents that could negatively influence ED, including Cymbalta (N=2), Escitalopram (N=2), Mirtazapin (N=1), Quetiapin (N=1), Saroten (N=1), Wellbutrin (N=2), Trittico (N=1), Venlafaxin (N=2), and Brintellix (N=2). The absence of medical record review is another limitation, as this study relied on patient self-report of sexual function and psychological parameters. Other limitations include not having information of mental health history in particular comorbid psychiatric illness of the ICD patient and their use of psychotherapy. Patients could potentially be in therapy for existing anxieties or posttraumatic stress disorder symptoms that have occurred since the implantation of the device, which may affect how the patient completes the survey.

## Conclusion

This study showed an association between severe ED and alexithymia and a moderating effect of ADNM-20 subscale preoccupation on the relationship between sexual dysfunction measured with IIEF and the alexithymia TAS-20 subscale externally oriented thinking. In light of the limited information available, it becomes essential to encourage further research in this area. Exploring the mechanisms that link ICD therapy and erectile function could yield valuable insights for clinicians, enabling more holistic patient care that considers not only the cardiovascular aspects but also the potential impact on psychological well-being and sexual health. As cardiovascular medicine continues to evolve, it is imperative to recognize and address the broader implications of treatments on various aspects of patients’ lives, including their intimate relationships and sexual function.

## Data Availability

The raw data supporting the conclusions of this article will be made available by the authors, without undue reservation.
